# Supplementary material for: The Chemical Signatures of Water Extract of Zingiber officinale Rosc
Source: Molecules. 2022 Nov 13;27(22):7818. doi: 10.3390/molecules27227818 (PMC9696620; doi:10.3390/molecules27227818)
Supplement: Supplementary file 1 [file molecules-27-07818-s001.zip › molecules-1971448-supplementary.pdf]

Supplementary Material

Supplement Table S1. Data of chemical substances database of *Zingiber officinale* Rosc.

Formula	Mass	Name
C7H16O	116.12	2-Heptanol
C6H13NO2	131.09	Isoleucine
C5H11NO3	133.07	Mesotrihydroxypiperidine
C10H14	134.11	p-Cymene
C10H16	136.13	alpha-Limonene
C10H16	136.13	beta-Phellandrene
C10H16	136.13	camphene
C10H16	136.13	D-Limonene
C10H16	136.13	alpha-Pinene
C10H16	136.13	Camphene
C10H16	136.13	1R,5R-(+)-alpha-Pinene
C10H16	136.13	Terpinolene
C10H16	136.13	4(10)-Thujene
C10H16	136.13	beta-Pinene
C7H5O3	137.02	Salicylic acid
C9H18O	142.14	2-Nonanone
C9H18O	142.14	Nonaldehyde
C10H22	142.17	Decane
C9H20O	144.15	2-Nonanol
C5H11NS2	149.03	Nereistoxin
C8H9NO2	151.06	2-(methylamino)benzoic acid
C10H16O	152.12	(Z)-Citral
C10H16O	152.12	Citral
C10H16O	152.12	(-)-Camphor
C10H16O	152.12	(E)-Citral
C10H18O	154.14	alpha-Linalool
C10H18O	154.14	1,8-Cineole
C10H18O	154.14	Geraniol
C10H20O	156.15	Decanal
C9H8NO2	162.06	Homovanillonitrile
C9H7O3	163.04	p-Coumaric acid
C6H11O5	163.06	2-Deoxy-D-glucose
C9H11NO2	165.08	phenylalanine
C11H22O	170.17	2-Undecanone
C10H9O3	177.06	3-Buten-2-on, 4-(2,5-dihydroxyphenyl)
C6H11O6	179.06	glucose
C6H7O7	191.02	Citric acid
C11H14O3	194.09	zingerone
C10H11O4	195.07	Dihydroferulic acid
C12H20O2	196.15	Geranyl acetate
C15H22	202.17	Curcumene
C11H7O4	203.03	7-Methoxycoumarin-6-aldehyde

C14H21N	203.17	2,6-Nonamethylene pyridine
C11H12N2O2	204.09	Tryptophan
C15H24	204.19	beta-Bisabolene
C15H24	204.19	beta-Curcumene
C15H24	204.19	beta-Farnesene
C15H24	204.19	Zingiberene
C15H24	204.19	Farnesene
C15H24	204.19	Bisabolene
C15H24	204.19	(E,E)-alpha-Farnesene
C15H24	204.19	Cedr-8-ene
C15H24	204.19	(-)-Germacrene D
C15H24	204.19	(+)-Cyclosativene
C14H21O	205.16	Benzenepentanol,α,α,4-trimethyl
C16H26	218.2	beta-Sesquiphellandrene
C14H20O2	220.15	(+)-1,5-Epoxy-nor-ketoguaia-11-ene
C15H24O	220.18	Shyobunone
C15H24O	220.18	Zingiberone
C13H17O3	221.12	Paradol
C14H21O2	221.15	Kobusone
C15H26O	222.2	Campherenol
C14H29NO	227.22	Halaminol A
C15H26O2	238.19	Curcumadiol
C16H22O2	246.16	Demethoxy-[6]-shogaol
C15H20O3	248.14	[4]-Shogaol
C16H24O2	248.18	3-Dihydro-[6]-demethoxyshogaol
C16H31NO	253.24	Palmitoleamide
C16H32O2	256.24	Palmitic acid
C16H22O3	262.16	Methy-[4]-shogaol
C16H24O3	264.17	Demethoxy-[6]-gingerol
C15H22O4	266.15	[4]-Gingerol
C15H24O4	268.17	[4]-Gingerdiol
C15H10O5	270.05	Galangin
C17H34O2	270.26	Methyl hexadecanate
C17H24O3	276.17	[6]-Shogaol
C17H26O3	278.19	[6]-Paradol
C18H17NO2	279.13	Hptaphylline
C16H24O4	280.17	Methyl-[4]-gingerol
C17H28O3	280.2	Dihydro-[6]-paradol
C16H25O4	281.18	1,2-benzendiol derivative 3(5-(3,5-bis(acetoxyl)-7-(4-hydroxy-3-methoxyphenyl)heptyl)-3-methoxy-)
C18H35NO	281.27	Oleamide
C17H22O4	290.15	Methyl-[6]-shogaol
C17H22O4	290.15	dehydro-6-gingerdione
C18H26O3	290.19	1-Dehydro-[6]-gingerdione
C17H24O4	292.17	6-Dehydro-[6]-gingerol
C17H24O4	292.17	[6]-Gingerdione

C17H24O4	292.17	1-Dehydro-[6]-gingerrol
C17H24O4	292.17	6-Hydroxy-[6]-shogaol
C17H24O4	292.17	dehydro-6-gingerol
C18H28O3	292.2	[7]-Paradol
C17H26O4	294.18	[6]-Gingerol
C17H28O4	296.2	[6]-Gingerdiol
C13H20N3O5	298.14	L-arabino-hept-2-enar-7-amic acid derivative
C18H34O3	298.25	Oxo-octadecenoic acid
C18H37NO2	299.28	Amino-octadecene-diol
C15H10O7	302.04	pentahydroxyflavone
C19H28O3	304.2	[8]-Shogaol
C19H30O3	306.22	[8]-Paradol
C17H24O5	308.16	Acetoxy-[4]-gingerol
C18H28O4	308.2	Methyl-[6]-gingerol
C18H28O4	308.2	Me-6-gingerol
C20H36O2	308.27	Labdene-diol
C17H26O5	310.18	3-Acetoxy-[4]-gingerdiol
C16H12O7	316.06	Sexangularetin
C19H26O4	318.18	1-Dehydro-[8]-gingerdione
C20H30O3	318.22	Methyl-[8]-shogaol
C20H30O3	318.22	(E)-8,17-Epoxylabd-12-ene-15,16-dial
C19H28O4	320.2	[8]-Gingerdione
C19H28O4	320.2	6-Hydroxy-[8]-shogaol
C19H28O4	320.2	dehydro-8-gingerol
C20H32O3	320.24	[9]-Paradol
C19H30O4	322.21	(S)-8-Gingerol
C19H32O4	324.23	[8]-Gingerdiol
C20H22O4	326.15	Gingerenone C
C18H33O5	329.23	Octadecenoic acid, -trihydroxy
C18H34O5	330.24	trihydroxy octadecenoic acid
C21H32O3	332.24	10-Shogaol
C19H26O5	334.18	dehydro-8-gingerdione
C21H34O3	334.25	[10]-Paradol
C19H28O5	336.19	Acetoxy-[6]-gingerol
C20H32O4	336.23	Methyl-[8]-gingerol
C20H32O4	336.23	Me-8-gingerol
C19H30O5	338.21	3-Acetoxy-[6]-gingerdiol
C20H22O5	342.15	1-(4-hydroxy-3-methoxyphenyl)-7-(3,4-dihydroxyphenyl)heptanone
C20H24O5	344.16	5-Hydroxy-1-(4-hydroxy-3-methoxyphenyl)-7-(4-hydroxyphenyl)-3-heptanone
C20H25O5	345.17	Rosmanol
C21H30O4	346.21	1-Dehydro-[10]-gingerdione
C21H30O4	346.21	dehydro-10-gingerdione
C21H32O4	348.23	e1 - Dehydro-3-dihydro-[10]-gingerdion
C21H32O4	348.23	[10]-Gingerdione

C21H32O4	348.23	6-Hydroxy-[10]-shogaol
C21H32O4	348.23	dehydro-10-gingerol
C20H30O5	350.21	Methyl acetoxy-[6]-gingerol
C21H34O4	350.25	[10]-Gingerol
C19H28O6	352.19	Diacetoxy-[4]-gingerdiol
C20H32O5	352.23	Methyl-3-acetoxy-[6]-gingerdiol
C21H36O4	352.26	[10]-Gingerdiol
C21H24O5	356.16	1,7bis-(4-Hydroxy-3-methoxyphenyl)-4-heptanone
C21H24O5	356.16	Gingerenone A
C21H24O5	356.16	Gingerenone A
C21H28O5	360.19	Cinerins
C23H36O3	360.27	[12]-Shogaol
C21H32O5	364.23	Acetoxy-[8]-gingerol
C22H36O4	364.26	Methyl-[10]-gingerol
C21H17O6	365.1	Diarylheptanoid
C20H30O6	366.2	Methyl diacetoxy-[4]-gingerdiol
C17H19O9	367.1	Feruloylquinic acid
C21H22O6	370.14	Dihydrocurcumin
C21H24O6	372.16	1,7bis-(4-Hydroxy-3-methoxyphenyl)-3,5-heptadione
C21H24O6	372.16	Tetrahydrocurcumin
C21H26O6	374.17	5-Hydroxy-1,7-bis(4-hydroxy-3-methoxyphenyl)-3-heptanone
C21H26O6	374.17	3,5-diHydroxy-1,7-bis(4-hydroxy-3-methoxyphenyl)-3-heptanone
C21H26O6	374.17	3-Acetoxy-5-hydroxy-1-(4-hydroxyphenyl)-7-(3,4-dihydroxyphenyl)heptane
C21H26O6	374.17	hexahydrocurcumin
C23H34O4	374.25	1-Dehydro-[12]-gingerdione
C23H34O4	374.25	dehydro-12-gingerdione
C21H27O6	375.18	Octahydrocurcumin
C23H36O4	376.26	3,5-diHydroxy-1,7-bis(4-hydroxy-3-methoxyphenyl)heptane
C23H36O4	376.26	[12]-Gingerdione
C23H36O4	376.26	dehydro-12-gingerol
C23H38O4	378.28	[12]-Gingerol
C21H32O6	380.22	Diacetoxy-[6]-gingerdiol
C14H23O12	383.12	Sucrose 6-acetate
C22H26O6	386.17	1-(4-Hydroxy-3,5-dimethoxyphenyl)-7-(4-hydroxy-3-methoxyphenyl)-heptanone
C22H26O6	386.17	Gingerenone B
C22H28O6	388.19	1,7bis-(4-Hydroxy-3-methoxyphenyl)-5-methoxy-3-heptanone
C19H32O8	388.21	12,13-Di-acetoxyl-1,4,6,11-eudesmanetetol
C21H25O7	389.16	3-Heptanone derivative(1-(3,4-dihydroxy-5-methoxyphenyl)-5-hydroxy-7-(4-hydroxy-3-methoxyphenyl-))
C21H26O7	390.17	5-Hydroxy-1-(3,4-dihydroxy-5-methoxyphenyl)-7-(4-hydroxy-3-methoxypenyl)-3-heptanone
C21H26O7	390.17	3-Acetoxy-5-hydroxy-1,7-bis(3,4-dihydroxyphenyl)heptane
C21H26O7	390.17	1,5-Epoxy-3-hydroxy-1-(3,4-dihydroxy-5-methoxyphenyl)-7-(4-hydroxy-3-methoxyphenyl)heptane

C23H36O5	392.26	Acetoxy-[10]-gingerol
C18H12N5O6	394.08	DPPH
C22H34O6	394.24	Methyl-diacetoxy-[6]-gingerdiol
C15H25O12	397.13	d-Glucopyranoside 2
C25H38O4	402.28	dehydro-14-gingerdione
C22H28O7	404.18	5-Hydroxy-1-(4-hydroxy-3,5-dimethoxyphenyl)-7-(4-hydroxy-3-methoxyphenyl)-3-heptanone
C22H28O7	404.18	3-Acetoxy-5-hydroxy-1-(4-hydroxy-3-methoxyphenyl)-7-(3,4-dihydroxyphenyl)heptane
C22H28O7	404.18	1,5-Epoxy-3-hydroxy-1-(4-hydroxy-3,5-dimethoxyphenyl)-7-(4-hydroxy-4-methoxyphenyl)heptane
C22H28O7	404.18	5?-Hydroxy-1-(4-hydroxy-3,5-dimethoxyphenyl)-7-(4-hydroxy-3-methoxyphenyl)-3-heptanone
C22H29O7	405.19	3,5-heptanediol derivative 1(1-(4-hydroxy-3,5-dimethoxyphenyl)-7-(4-hydroxy-3-methoxyphenyl-))
C22H30O7	406.2	3,5-dihydroxy-1-(4-hydroxy-3,5-dimethoxyphenyl)-7-(4-hydroxy-3-methoxyphenyl)heptane
C23H36O6	408.25	Diacetoxy-[8]-gingerdiol
C23H28O7	416.18	3,5-Diacetoxy-1-(3,4-dihydroxyphenyl)-7(4-hydroxyphenyl)heptane
C23H30O7	418.2	3-Acetoxy-5-hydroxy-1,7-bis(4-hydroxy-3-methoxyphenyl)heptane
C22H28O8	420.18	3-Acetoxy-5-hydroxy-1-(3,4-dihydroxyphenyl)-7-(3,4-dihydroxy-5-methoxyphenyl)heptane
C24H38O6	422.27	Methyl diacetoxy-[8]-gingerdiol
C24H29O7	429.19	3,5-heptanediol derivative 1(1-(4-hydroxy-3-methoxyphenyl)-7-(4-hydroxyphenyl)-)
C24H30O7	430.2	3,5-Diacetoxy-1-(4-hydroxy-3-methoxyphenyl)-7-(4-hydroxyphenyl)heptane
C23H28O8	432.18	3,5-Diacetoxy-1,7-bis(3,4-dihydroxyphenyl)heptane
C23H28O8	432.18	3-Acetoxy-1,5-epoxy-1-(3,4-dihydroxy-5-methoxyphenyl)-7-(4-hydroxy-3-methoxyphenyl)heptane
C27H28O5	432.19	Galanganol C
C30H40O2	432.3	Citraurin beta
C23H29O8	433.19	1,2-benzendiol derivative 1(5-(5-(acetoxyl)-3-hydroxy-7-(4-hydroxy-3-methoxyphenyl)heptyl)-3methoxy-)
C23H30O8	434.19	3-Acetoxy-5-hydroxy-1-(3,4-dihydroxy-5-methoxyphenyl)-7-(4-hydroxy-3-methoxyphenyl)heptane
C23H32O8	436.21	3,5-diHydroxy-1,7-bis(4-hydroxy-3,5-dimethoxyphenyl)heptane
C25H40O6	436.28	Diacetoxy-[10]-gingerdiol
C24H29O8	445.19	2H-Pyran-4-ol derivative(tetrahydro-2-(4-hydroxy-3,5-dimethoxyphenyl)-)
C24H30O8	446.19	3,5-Diacetoxy-1-(3,4-dihydroxyphenyl)-7-(4-hydroxy-3-methoxyphenl)heptane
C24H32O8	448.21	3-Acetoxy-5-hydroxy-1-(4-hydroxy-3-methoxyphenyl)-7-(4-hydroxy-3,5-dimethoxyphenyl)
C24H32O8	448.21	3-Acetoxy-5-hydroxy-1-(3,4-dihydroxy-5-methoxyphenyl)heptane

C23H30O9	450.19	3-Acetoxy-5-hydroxy-1,7-bis(3,4-dihydroxy-5-methoxyphenyl)heptane
C26H42O6	450.3	Methyl diacetoxy-[10]-gingerdiol
C27H36O6	456.25	Meso-3,5-diacetoxy-1,7-bis-(4-hydroxy-3-methoxyphenyl) heptane
C25H32O8	460.21	3,5-Diacetoxy1,7-bis(4-hydroxy-3-methoxyphenyl)heptane
C24H29O9	461.18	1,2-benzendiol derivative 2(5-(3,5-bis(acetoxyl)-7-(3,4-dihydroxyphenyl)heptyl)-3-methoxy-)
C24H30O9	462.19	3,5-Diacetoxy-1-(3,4-dihydroxyphenyl)-7-(3,4-dihydroxy-5-methoxyphenyl)heptane
C25H31O9	475.2	1,2-benzendiol derivative 4(5-(3,5-bis(acetoxyl)-7-(4-hydroxy-3-methoxyphenyl)heptyl)-3-methoxy-)
C25H32O9	476.2	3,5-Diacetoxy-1-(4-hydroxy-3-methoxyphenyl)-7-(3,4-dihydroxy-5-methoxyphenyl)heptane
C18H29O15	485.15	d-Fructofuranose
C26H34O9	490.22	3,5-Diacetoxy-1-(4-hydroxy-3,5-dimethoxyphenyl)-7-(4-hydroxy-3-methoxyphenyl)heptane
C25H32O10	492.2	3,5-Diacetoxy-1,7-bis(3,4-dihydroxy-5-methoxyphenyl)heptane
C21H40NO12	498.26	d-Glucopyranoside 2
C26H34O10	506.22	3,5-Diacetoxy-1-(3,4-dihydroxy-5-methoxyphenyl)-7-(4-hydroxy-3,5-dimethoxyphenyl)heptane
C25H24O12	516.13	Diacetylafzelin
C18H24N6O6S4	548.06	ABTS
C26H28O13	548.15	Hemsleyanoside
C22H40NO15	558.24	d-Glucopyranoside 3
C21H35O 17	559.19	d-Arabinofuranoside
C26H28O14	564.15	Kaempferol rhamnoside xyloside
C32H22O10	566.12	Ginketin
C27H30O16	610.15	kaempferol diglucoside
C27H30O16	610.15	Rutin
C30H27O14	611.14	Delphinidin coumaroyl glucoside/Delphinidin-3-O-(6-p-coumaroyl)glucoside
C24H35O18	611.18	L-Glycero-manno-heptopyranose
C25H31N4O14	611.18	A-arabitol derivative
C24H39O20	647.2	d-Glucopyranoside 1
C33H58O14	678.38	Gingerglycolipid A/B
C32H44N7O10	686.32	Peptide 1
C27H45O22	721.24	d-Glucopyranoside 1
C34H47N8O10	727.34	Protein 1
C35H43N12O6	727.34	Alaninamid derivative(N-(4-(((2,4-diamino-6-pteridinyl)methyl)methylamino)benzoyl)-l-?-glutamyl-)
C45H72O17	884.48	Zingiberoside A3
C41H28O27	952.08	Geraniin
C55H88O21	1084.6	Cynanuriculoside A
